# The association of physical activity with kidney function risk among adults with long working hours

**DOI:** 10.3389/fendo.2024.1415713

**Published:** 2024-09-30

**Authors:** Tenglong Yan, Subat Nabi, Xiaodong Liu, Bangzhao Zeng, Xin Song, Xiaowen Ding, Binshuo Hu

**Affiliations:** ^1^ Beijing Institute of Occupational Disease Prevention and Control, Beijing, China; ^2^ School of Public Health, Peking University, Beijing, China; ^3^ School of Public Health, North China University of Science and Technology, Tangshan, China; ^4^ School of Public Health and the Beijing Key Laboratory of Environmental Toxicology, Capital Medical University, Beijing, China

**Keywords:** long working hours, estimated glomerular filtration rate (eGFR), chronic kidney diseases, physical activity (PA), modification

## Abstract

**Introduction:**

Long working hours are likely associated with the decreased of kidney function, while physical activity (PA) was linked to improvements in kidney function. However, whether PA can offset the negative impact of long working hours on kidney function was unclear, which is the focus of this study.

**Methods:**

A cross-sectional study was conducted. Three approaches were adopted to distinguish the association between long working hours and regular working hours. Moderate to vigorous physical activity (PA) was assessed by a structured questionnaire. eGFR and chronic kidney disease (CKD) or not were utilized to evaluate the kidney function. Linear and logistic regression analyses were conducted to assess the association between weekly working hours, PA, and kidney function.

**Results:**

A total of 18,431 adults were enrolled in this study, including 9981 males (54.2%) and 8450 females (45.8%). The average eGFR was (99.54 ± 17.55 mL/min/1.73 m^2^). The people worked more than 40 h/wk (98.89 ± 17.06 mL/min/1.73 m^2^) had lower eGFR compared to those worked less than 40 h/wk (99.93 ± 17.83 mL/min/1.73 m^2^) (*p* < 0.05). Individuals working longer hours exhibited lower eGFR (*β* = -0.772, 95% *CI*: -1.241, -0.303, for > 40 h/wk compared to working ≤ 40 h/wk). Engagement in moderate to vigorous PA was associated with higher eGFR values (*β* = 1.159, 95% *CI*: 0.699, 1.619) compared to low PA (< 150 minutes/wk), but this association did not reach statistical significance for the prevalence of CKD. Furthermore, PA was insufficient to reverse the decline of eGFR related to prolonged working hours.

**Discussion:**

Prolonged working hours were associated with a decline in eGFR, while PA was found to have a protective effect on kidney function. However, PA alone may not fully mitigate the negative impact of prolonged working hours on renal health. More robust measures to protect renal function should be implemented to mitigate the damage caused by prolonged working hours.

## Graphical Abstract

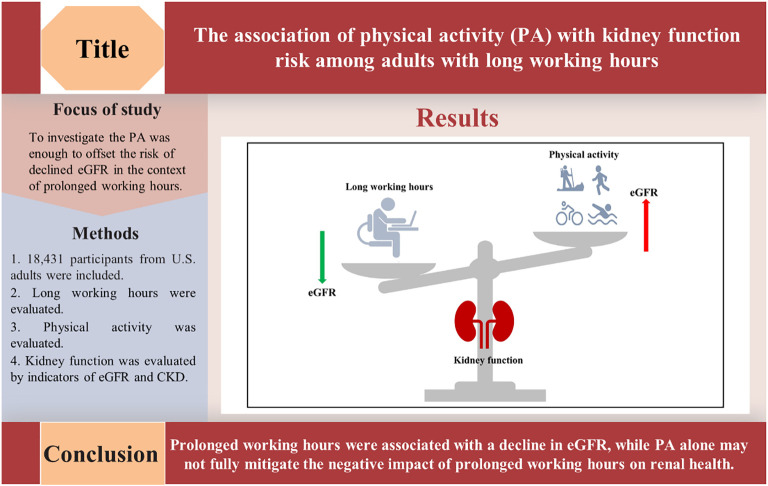

## 1 Introduction

Extended working hours were on the rise in contemporary society, prompting apprehensions regarding the potential ramifications on diverse facets of health. The mean weekly working time worldwide stood at approximately 43 hours, with approximately 36.1% of workers clocking in more than 48 h/wk in 2014 reported by the International Labour Organization (ILO) (1). Based on population-based surveys and the Sixth European Working Conditions Survey, the prevalence of working 48 or more hours per week was notably high in 2010, with 19% of Americans working such hours (2). In comparison, in 2015, 15% of Europeans reported working similar hours (2). Consistent exposure to prolonged work hours has been consistently associated with an array of safety and health risks, such as cardiovascular diseases, hypertension, depression, and occupational injury (3–5), while renal function stands out as a vital parameter, given its pivotal role in maintaining metabolic (6) balance and waste excretion. Some evidences from Asia showed that long working hours were associated with decreased kidney function and increased prevalence of chronic kidney disease (CKD) (7, 8), while whether this pattern existed in other ethnic groups remains unclear.

Epidemiological studies indicated a global CKD prevalence ranging from 8% to 16%, affecting approximately 850 million individuals globally (9). In 2017, approximately 1.2 million (with a 95% uncertainly interval ranging from 1.2 to 1.3 million) individuals succumbed to CKD worldwide, leading to 35.8 million (with a 95% uncertainly interval ranging from 33.7 to 38.0 million) disability-adjusted life-years (DALYs) (10). Notably, CKD was a major risk factor for cardiovascular diseases, the leading cause of death globally, amplifying its association on public health (10, 11). Given its multifactorial etiology and complex pathophysiology, effective strategies for the prevention and management of CKD required a comprehensive approach, encompassing early detection, risk factor modification, and timely interventions to mitigate disease progression. Understanding the risk factors of CKD was crucial for informing public health policies and strategies aimed at reducing its burden and improving the overall health outcomes of affected individuals globally, while long working hours probably be a potential risk factor for CKD.

Physical activity (PA) played a crucial role in maintaining overall health and preventing various diseases. Numerous studies have consistently demonstrated the beneficial effects of regular PA on reducing the risk of chronic conditions such as depression, cardiovascular disease, diabetes, obesity, and certain cancers (12–15). Regarding CKD, emerging evidence suggested that PA probably be also play a protective role in kidney health (16–19). We therefore considered it necessary to evaluate the relationship of kidney function with long working hours, as well as whether PA has sufficiently protective effect on it, to reveal the adverse effects of long working hours, draw attention to the harms of prolonged work periods, and ultimately reduce working hours. As estimated glomerular filtration rate (eGFR) testing was affordable and widely accessible globally, diminished eGFR may serve as a valuable indicator for predicting kidney disease and its implications (20). The hypothesis of this study is that long working hours are associated with a decline in kidney function, while physical activity is associated with improvements in kidney function, and physical activity may be able to offset this damage. The objective of this study was to explore the correlation between weekly work hours and declining kidney function, as measured by eGFR, and further evaluate the association of PA, among the long working individuals in U.S.

## 2 Materials and methods

### 2.1 Participants and study design

**Figure 1:**
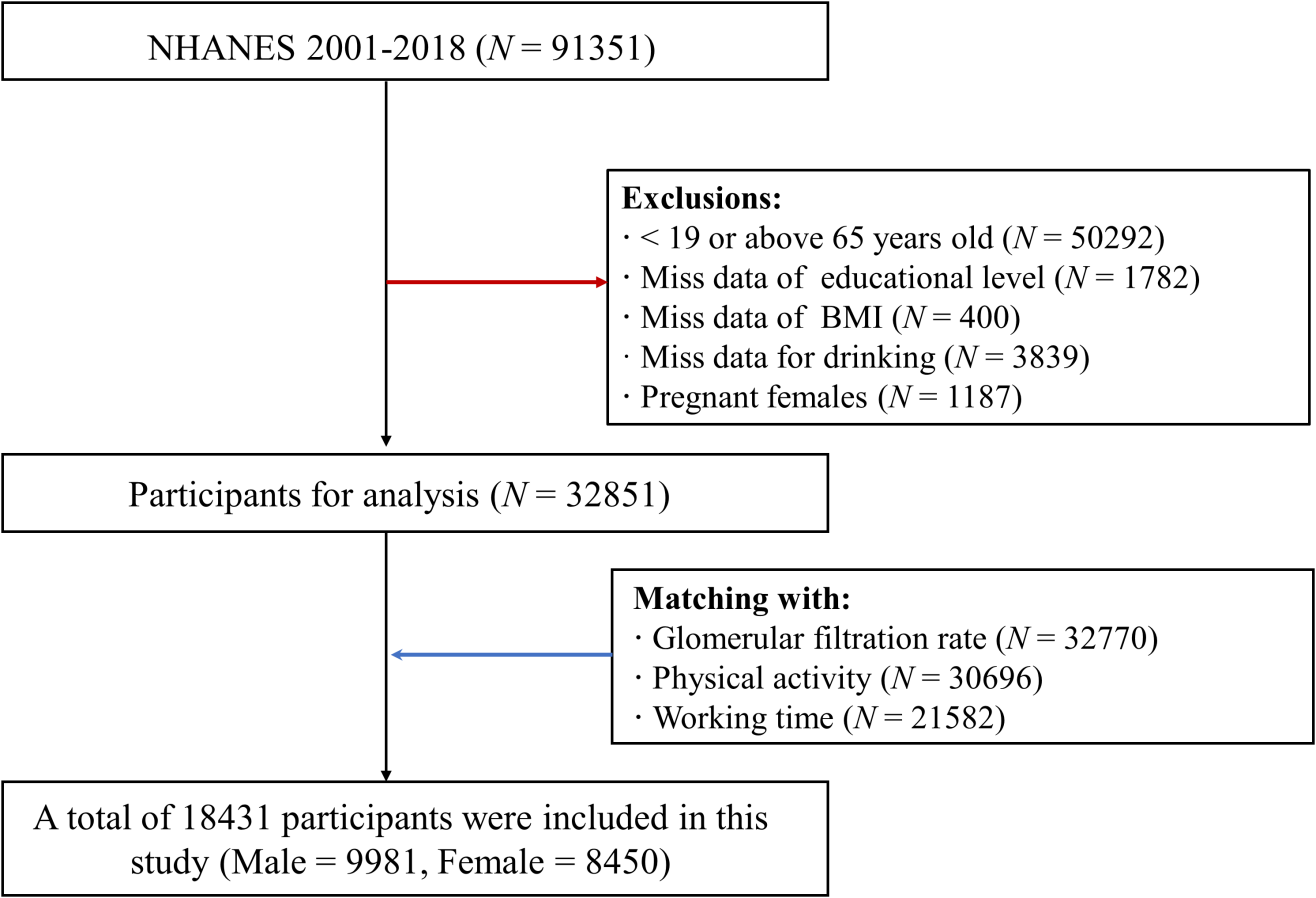
Participants inclusion and exclusion flow chart.

The research leveraged the dataset from the NHANES, a representative cross-sectional study conducted in the U.S. The NHANES study protocol has been approved by the National Center for Health Statistics (NCHS) Institutional Research Ethics Review Board (ERB). In this study, data was utilized in the nine cycle years, including 2001 – 2002, 2003 - 2004, 2005 - 2006, 2007 - 2008, 2009 - 2010, 2011 - 2012, 2013 – 2014, 2015 - 2016, and 2017 - 2018. A total of 91,351 individuals were enrolled in primary. And 50,529 participants were excluded for < 19 or above 65 years old. 1748, 400, and 3839 individuals were excluded for missing data of educational level, BMI, and drinking information, respectively. And 1187 pregnant females were also excluded. Furthermore, based on participants’ code, demographic and sociological information with data on eGFR, PA, and working time was matched. In final, 18,431 individuals were included, consistent of 9981 males (54.2%) and 8450 (45.8%) females (**Figure 1**).

### 2.2 Working hours

The assessment of weekly working hours was conducted via a questionnaire, available at https://wwwn.cdc.gov/nchs/nhanes/Default.aspx. All the participants were questioned “How many hours did [you/SP] work last week at all jobs or businesses [OCQ180]”. Due to the inconsistent classification criteria for long working hours among different international organizations, while there was no research to show which is better, three distinct categorizations were employed for weekly working hours in this study (4, 21). Category 1: ≤ 40 h/wk and > 40 h/wk; Weekly working hours category 2: ≤ 48 h/wk and 48 h/wk; Weekly working hours category 3: ≤ 30 h/wk, 31 - 40 h/wk, 41 - 48 h/wk, 49 – 54 h/wk, and ≥ 55 h/wk.

### 2.3 Kidney function

The blood for all the participants were collected by trained nurses, centrifuged and shipped to – 80 °C environment until test. The Jaffe rate method was used to analyzed serum creatine. Furthermore, according to the indicator of serum creatine, age, sex, and race/ethnicity, the Chronic Kidney Disease Epidemiology Collaboration (CKD-EPI) equation was applied for eGFR estimation (22). Additionally, individuals whose eGFR was below 60 mL/min/1.73 m² were classified as cases of CKD (23).

### 2.4 Physical activity (PA)

Within the NHANES dataset, the moderate to vigorous PA levels in leisure-time were assessed using the following questionnaire items: PADLEVEL (Activity level), PADTIMES (# of times did activity in the past 30 days), which included activity type and time. The moderate intensity to vigorous PA have a set of fixed and clear evaluation methods and were widely accepted by most scholars, including swimming, walking, bicycling et al. (24, 25) PADDURAT (Average duration of activity in minutes). Utilizing this data, the moderate to vigorous PA was computed as PADTIMES (# of times did activity in the past 30 days) × PADDURAT (Average duration of activity in minutes). We defined engagement in moderate to vigorous PA for less than 150 minutes per week in leisure-time as high-risk group, whereas participation in moderate to vigorous PA for 150 minutes or more per week was categorized as low-risk group (24).

### 2.5 Covariates

The demographic characteristics extracted from the NHANES survey encompassed a range of variables, including sex, age, race/ethnicity (categorized as Mexican American, Other Hispanic, Non-Hispanic White, Non-Hispanic Black, and Other race), body mass index (BMI), smoking habits, alcohol consumption habits, and hypertension. Age was stratified into two groups: 20-45 years and 46-65 years. The race/ethnicity was further subdivided into two group: White or other race and Black groups, as the CKD-EPI equation calculates the eGFR separately based on whether the individual belongs to the Black race (22). BMI was computed as weight (in kilograms) divided by height squared (in meters), and categorized into three levels: < 25, 25 to 30, and ≥ 30 kg/m^2^. Participants were identified as drinkers if they reported consuming alcohol more than 14 g (women) or 28 g (men). Individuals were defined as smoker if they reported smoking more than 100 cigarettes in their lifetime. In this study, hypertension was defined based on systolic blood pressure, diastolic blood pressure, and physician diagnosis of hypertension. Participants were considered to have hypertension (26) if their (1) systolic blood pressure was greater than 140 mmHg, (2) or if the diastolic blood pressure was greater than 90 mmHg, (3) or if they had received a diagnosis of hypertension from a physician. Meeting any one of these criteria led to classification as hypertensive.

### 2.6 Statistical analysis

Statistical analyses were conducted using SPSS 26.0 (SPSS Inc., Chicago, USA) for Windows and R software (version 4.0.4; R Core Team). Continuous variables were presented as mean ± standard deviation (*SD*), while categorical parameters were expressed as *n* (%). To compare differences of eGFR between different sub-groups participants, Student’s *t*-test or analysis of variance (ANOVA) were employed for continuous variables, while the chi-square test was utilized for categorical variables. Linear and logistic regression analyses were performed to assess the association of longer working hours with the decreased of eGFR and the risk of CKD, with three categories of working hours serving as the reference category, respectively. To ensure the robustness of the results, three levels of adjustment were applied. Initially, the crude model was analyzed, followed by Model 1, which was adjusted for age, gender, BMI, and race/ethnicity, related with the kidney function previously (27, 28). Model 2 further adjusted for smoking & alcohol habits, and hypertension, which were reported related with kidney function previously (28–30). Statistical significance was defined as *α* < 0.05 for two-tailed *p*-values.

## 3 Results

### 3.1 The general demographic characteristics of participants

The general demographic characteristics of participants of the 18,431 participants included in the study was outlined in **Table 1**. The study consisted of 9,981 (54.2%) males and 8,450 (45.8%) females, providing a balanced representation of both sexes. The participants were categorized into two age groups. The majority, comprising 11,227 (60.9%), fell within the 20-45 years age range, while 7,204 (39.1%) were in the 46-65 years age category. There were 14707 (79.8%) white or other race individuals and 3724 (20.2%) included in this study. The BMI categories were delineated as follows: 31.7% (*n* = 5,802) had a BMI of <no><</no> 25 kg/m^2^, 36.2% (*n* = 6,638) fell within the 25 to 30 kg/m^2^ range, and 32.1% (*n* = 5,882) had a BMI ≥ 30 kg/m^2^. Smoking was reported by 3,696 of participants (20.1%), while the majority, 14,735 (79.9%), were non-smokers. Approximately 29.1% of participants (*n* = 5,361) reported alcohol consumption, with the remaining 70.9% (*n* = 13,070) abstaining. Hypertension was present in 28.0% of participants (*n* = 5,153), while 72.0% (*n* = 13,278) did not exhibit hypertension. A breakdown of physical activity levels revealed that 36.2% (*n* = 6,672) engaged in more than 150 minutes per week, whereas 63.8% (*n* = 11,759) participated in physical activity for less than 150 minutes per week. Participants were divided into two categories based on their weekly working hours. In the first category, 62.2% (*n* = 11,471) worked 40 hours or fewer per week, while 37.8% (*n* = 6,960) worked more than 40 hours. In the second category, working hours ranged from ≤ 48 h/wk to > 48 h/wk. The third category detailed working hours from ≤ 30 h/wk to ≥ 55 h/wk.

**Table 1:** The general demographic characteristics and kidney function of the participants.

Characteristics	All participants	eGFR (mL/min/1.73 m^2^)		CKD case (eGFR < 60mL/min/1.73 m^2^)		
	*n (%)*	Mean ± SD	*p*-value ^a^	Yes *n* (%)	No *n* (%)	*p*-value ^b^
All participants	18431 (100.0)	99.54 ± 17.55	—	283 (1.5)	18148 (98.5)	—
Sex			0.279			0.293
Male	9981 (54.2)	99.66 ± 17.90		162 (1.6)	9819 (98.4)	
Female	8450 (45.8)	99.38 ± 17.13		121 (1.4)	8329 (98.6)	
Age category			< 0.001^*^			< 0.001^*^
20 - 45 years old	11227 (60.9)	106.23 ± 15.81		35 (0.3)	11192 (99.7)	
46 - 65 years old	7204 (39.1)	89.11 ± 14.83		248 (3.4)	6956 (96.6)	
Race/ethnicity			< 0.001^*^			0.569
White or other	14707 (79.8)	98.18 ± 15.96		222 (1.5)	14485 (98.5)	
Black	3724 (20.2)	104.90 ± 21.95		61 (1.6)	3663 (98.4)	
Body mass index (BMI)			< 0.001^*^			< 0.001^*^
< 25 kg/m^2^	5802 (31.7)	101.56 ± 17.03		58 (1.0)	5744 (99.0)	
25 to 30 kg/m^2^	6638 (36.2)	98.00 ± 17.12		104 (1.6)	6534 (98.4)	
≥ 30 kg/m^2^	5882 (32.1)	99.22 ± 18.33		119 (2.0)	5763 (98.0)	
Smoke			< 0.001^*^			0.001^*^
Yes	3696 (20.1)	102.47 ± 16.65		35 (0.9)	3661 (99.1)	
No	14735 (79.9)	98.80 ± 17.69		248 (1.7)	14487 (98.3)	
Alcohol			0.189			0.136
Yes	5361 (29.1)	99.27 ± 17.07		71 (1.3)	5290 (98.7)	
No	13070 (70.9)	99.64 ± 17.74		212 (1.6)	12858 (98.4)	
Hypertension			< 0.001^*^			< 0.001^*^
Yes	5153 (28.0)	93.82 ± 17.93		181 (3.5)	4972 (96.5)	
No	13278 (72.0)	101.76 ± 16.89		102 (0.8)	13176 (99.2)	
Physical activity			0.065			0.001^*^
< 150 min/wk	11759 (63.8)	99.36 ± 17.61		208 (1.8)	11551 (98.2)	
≥ 150 min/wk	6672 (36.2)	99.85 ± 17.43		75 (1.1)	6597 (98.9)	
Weekly working hours category 1			< 0.001^*^			0.987
≤ 40 h/wk	11471 (62.2)	99.93 ± 17.83		176 (1.5)	11295 (98.5)	
> 40 h/wk	6960 (37.8)	98.89 ± 17.06		107 (1.5)	6853 (98.5)	
Weekly working hours category 2			< 0.001^*^			0.792
≤ 48 h/wk	13930 (75.6)	99.80 ± 17.68		212 (1.5)	13718 (98.5)	
> 48 h/wk	4501 (24.4)	98.71 ± 17.11		71 (1.6)	4430 (98.4)	
Weekly working hours category 3			< 0.001^*^			0.980
≤ 30 h/wk	3494 (19.0)	100.43 ± 18.56		55 (1.6)	3439 (98.4)	
31 - 40 h/wk	7977 (43.3)	99.71 ± 17.49		121 (1.5)	7856 (98.5)	
41 - 48 h/wk	2459 (13.3)	99.23 ± 16.95		36 (1.5)	2423 (98.5)	
49 – 54 h/wk	1937 (10.0)	98.12 ± 17.45		27 (1.5)	1810 (98.5)	
≥ 55 h/wk	2664 (14.5)	99.54 ± 17.55		44 (1.7)	2620 (98.3)	

^*^ *p*-value < 0.05. ^a^analysis of variance. ^b^Chi square test.

### 3.2 Kidney function

The kidney function across different groups of the participants were showed in **Table 1**. For all participants, the mean eGFR was (99.54 ± 17.55) mL/min/1.73 m^2^. Out of the total, 283 participants (1.5%) were identified as chronic CKD cases with eGFR < 60 mL/min/1.73 m^2^, while 18,148 participants (98.5%) had an eGFR above this threshold. No significant difference was observed in eGFR between males (99.66 ± 17.90 mL/min/1.73 m^2^ and females (99.38 ± 17.13 mL/min/1.73 m²). The prevalence of CKD cases among males and females was 1.6% and 1.4%, respectively. A substantial age-related variation in mean eGFR was found (*p* < 0.05). Participants aged 20-45 years had a higher mean eGFR (106.23 ± 15.81 mL/min/1.73 m²) and a lower CKD prevalence (0.3%) compared to those aged 46-65 years (eGFR 89.11 ± 14.83 mL/min/1.73 m^2^; CKD prevalence 3.4%). The eGFR of black man (104.90 ± 21.95 mL/min/1.73 m²) was higher than that of white or other race individuals (98.18 ± 15.96 mL/min/1.73 m²) (*p* < 0.05), while the CKD prevalence was without significance between the two ethnic groups. BMI categories exhibited a significant association on both mean eGFR (*p* < 0.05) and CKD prevalence. Lower mean eGFR and higher CKD prevalence were associated with higher BMI categories. Participants who smoked demonstrated a significantly higher mean eGFR (102.47 ± 16.65 mL/min/1.73 m^2^) and a lower CKD prevalence (0.9%) compared to non-smokers. No significant differences were observed in mean eGFR or CKD prevalence between participants who consumed alcohol and those who did not. Participants with hypertension had a lower mean eGFR (93.82 ± 17.93 mL/min/1.73 m²) and a higher CKD prevalence (3.5%) compared to those without hypertension. Higher physical activity levels were associated with a lower CKD prevalence, although the mean eGFR difference was not statistically significant. The results showed that the eGFR was (98.87 ± 17.06 mL/min/1.73 m^2^) in low PA group and (98.94 ± 17.03 mL/min/1.73 m^2^) in high PA group, which showed that in the context of long working hours, increased physical activity did not improve kidney function.

### 3.3 Associations of kidney function and long working hours

Weekly working hours significantly influenced kidney function. Individuals with longer working hours showed lower eGFR, although the CKD prevalence was not statistically significant (**Table 1**). The linear regression was performed in three levels models, which all indicated that individuals working longer hours tend to exhibit lower eGFR, after adjust three series of confounder factors (**Table 2**). After adjust variables of sex, age, race/ethnicity, BMI category, smoking and alcohol habits, and hypertension, working hours > 40 h/wk was negatively associated with decreased eGFR compared to those ≤ 40 h/wk [*β* = -0.772, 95% *CI* (-1.241, -0.303)]. Weekly working hours > 48 h/wk was negatively associated with decreased eGFR compared to those ≤ 48 h/wk [*β* = -0.838 (-1.365, -0.310)]. And the regression analysis of categorized working hours into five groups, further indicated a significant association between longer working hours and a decrease in eGFR (*p* trend < 0.05). The estimated associations of CKD across different working hour categories were further analyzed using three levels multiple logistic regression analysis (**Supplementary Table 1**). No significant difference was found in all the analysis.

**Table 2:** Estimated association of eGFR across long working hours performed by linear regression (*β*, 95% *CI*).

	Crude model	Model 1	Model 2
Weekly working hours category 1			
≤ 40 h/wk	Ref.	Ref.	Ref.
> 40 h/wk	-1.034 (-1.557, -0.512) ^*^	-0.979 (-1.447, -0.511) ^*^	-0.772 (-1.241, -0.303) ^*^
Weekly working hours category 2			
≤ 48 h/wk	Ref.	Ref.	Ref.
> 48 h/wk	-1.095 (1.685, -0.506) ^*^	-0.901 (-1.431, -0.372) ^*^	-0.838 (-1.365, -0.310) ^*^
Weekly working hours category 3			
≤ 30 h/wk	Ref.	Ref.	Ref.
31 - 40 h/wk	-0.718 (-1.415, -0.021) ^*^	-0.553 (-1.171, 0.064)	-0.575 (-1.190, 0.390)
41 - 48 h/wk	-1.197 (-2.102, -0.292) ^*^	-1.140 (-1.945, -0.336) ^*^	-1.226 (-2.027, -0.425) ^*^
49 – 54 h/wk	-2.305 (-3.295, -1.314) ^*^	-1.949 (-2.832, -1.067) ^*^	-1.867 (-2.745, -0.989) ^*^
≥ 55 h/wk	-1.313 (-2.197, -0.429) ^*^	-1.077 (-1.873, -0.280) ^*^	-1.075 (-1.867, -0.283) ^*^
*p* trend	< 0.001 ^*^	< 0.001 ^*^	< 0.001 ^*^

^*^ *p*-value < 0.05. Ref., Reference. Model 1. Sex, age, race/ethnicity, and BMI were adjusted. Model 2. Smoking & alcohol habits, and hypertension were further adjusted.

### 3.4 Associations of kidney function and physical activity

The association of kidney function and PA was tested by linear regression analyses were showed in **Table 3**. Individuals who engaged in 150 minutes or more of PA per week showed a higher eGFR values (*β* = 1.159, 95% *CI*: 0.699, 1.619). However, this association did not reach statistical significance for the prevalence of CKD (*OR* = 0.820, 95% *CI*: 0.625, 1.076) (**Supplementary Table 1**).

**Table 3:** Estimated association of eGFR and CKD across physical activity performed by linear regression (*β*, 95% *CI*).

Physical activity	eGFR (mL/min/1.73 m^2^)	CKD case (eGFR < 60mL/min/1.73 m^2^)
< 150 min/wk	Ref.	Ref.
≥ 150 min/wk	1.159 (0.699, 1.619) ^*^	0.820 (0.625, 1.076)

^*^ *p*-value < 0.05. Weekly working hours category, sex, age, race/ethnicity, BMI, smoking & alcohol habits, and hypertension were further adjusted.

### 3.5 Modification of PA on kidney function and working hours

The estimated associations of eGFR across different weekly working hours categories were analyzed in relation to PA time (**Table 4**). Adjustment for sex, age, race/ethnicity, BMI, smoking and alcohol habits, and hypertension, the results indicated that the decreased eGFR was more with individuals weekly working hours > 40 h/wk than those weekly working hours ≤ 40 h/wk. And the decreased of eGFR [*β* = -0.874, 95% *CI*: (-1.460, -0.288)] was less in the group of < 150 min/wk than those of ≥ 150 min/wk [*β* = -1.020, 95% *CI*: (-1.799, -0.242)]. The decreased eGFR was more with individuals weekly working hours > 48 h/wk than those weekly working hours ≤ 48 h/wk. And the decreased of eGFR [*β* = -0.813, 95% *CI*: (-1.471, -0.154)] was less in the group of < 150 min/wk than those of ≥ 150 min/wk [*β* = -0.911, 95% *CI*: (-1.790, -0.032)]. There were significant found in the other working hour category of eGFR. The estimated associations of CKD across different working hour categories and PA time were further analyzed using logistic regression analysis (**Supplementary Table 2**). No significant difference was found in all the analysis.

**Table 4:** Estimated association of eGFR across different weekly working hours category by physical activity time (*β*, 95% *CI*).

Physical activity time	Participants *n* (%)	*β* (95% *CI*)	*p*-value
< 150 min/wk			
≤ 40 h/wk	7352 (62.5)	Ref.	—
> 40 h/wk	4407 (37.5)	-0.874 (-1.460, -0.288)	0.003^*^
≥ 150 min/wk			
≤ 40 h/wk	4119 (61.7)	Ref.	—
> 40 h/wk	2553 (38.3)	-1.020 (-1.799, -0.242)	0.010^*^
< 150 min/wk			
≤ 48 h/wk	8897 (75.7)	Ref.	—
> 48 h/wk	2862 (24.3)	-0.813 (-1.471, -0.154)	0.016^*^
≥ 150 min/wk			
≤ 48 h/wk	5033 (75.4)	Ref.	—
> 48 h/wk	1639 (24.6)	-0.911 (-1.790, -0.032)	0.042^*^
< 150 min/wk			
≤ 30 h/wk	2141 (18.2)	Ref.	—
31-40 h/wk	5211 (44.3)	-0.489 (-1.263, 0.285)	0.215
41-48 h/wk	1545 (13.1)	-1.084 (-2.096, -0.071)	0.036^*^
49-54 h/wk	1156 (9.8)	-1.853 (-2.960, -0.745)	0.001^*^
≥ 55 h/wk	1664 (14.5)	-0.927 (-1.924, 0.070)	0.068
≥ 150 min/wk			
≤ 30 h/wk	1353 (20.3)	Ref.	—
31-40 h/wk	2766 (41.5)	-0.838 (-1.850, 0.175)	0.105
41-48 h/wk	914 (13.7)	-1.475 (-2.784, -0.165)	0.027^*^
49-54 h/wk	681 (10.2)	-1.969 (-3.410, -0.528)	0.007^*^
≥ 55 h/wk	958 (14.4)	-1.435 (-2.742, -0.128)	0.031^*^

^*^ *p*-value < 0.05. Ref., Reference. Sex, age, race/ethnicity, BMI, Smoking & alcohol habits, and hypertension were adjusted.

## 4 Discussion

This study provided insights into the complex interplay between PA, prolonged working hours, and kidney function among a large sample of participants. We observed that long working hours was associated with decreased kidney function, while PA levels, demonstrated a potential positive association with eGFR. However, when combined with long working hours, PA seems not to offset the adverse association on kidney function.

This study revealed a negative association between prolonged working hours and eGFR among U.S. adults for the first time. These findings were consistent with previous studies suggesting that extended working hours may influence kidney function among other regions. For every additional hour worked per week, there was a corresponding decrease of 0.057 mL/min/1.73 m^2^ in eGFR among individuals who worked more than 52 hours per week in the Korean population (7). The mechanism behind this association remains unclear, but sporadic studies suggested that the elevation of serum uric acid induced by stress from prolonged working hours probably be linked to the decreased of kidney function (31).

A wealth of evidence has reported that individuals who engaged in regular leisure-time PA have a lower risk of CKD compared to those who did not or engage in minimal PA (16–19). Moreover, enhancing PA has been shown to enhance symptom management, aerobic capacity, muscular strength, physical performance, cardiovascular health, and quality of life (17). In addition, evidences showed that occupational PA were found to increase the risk of poor self-rated health, while leisure-time PA was reverse (32). Furthermore, “weekend warrior” and other leisure-time PA were found both sufficient to reduce all-cause, CVD, and cancer mortality risk (33). The evidences supported the positive value of leisure-time PA. Given the prevalence of kidney inflammation in renal injury (34), leading to fibrosis and irreversible loss of function (35). The mechanism behind exercise’s potent anti-inflammatory effect involved the reduction of visceral fat, leading to decreased secretion of pro-inflammatory adipokines (36), and increased levels of anti-inflammatory cytokines, such as interleukin-6 (IL-6) released from contracting skeletal muscle (36). Elevated IL-6 levels have been shown to slow the increase in tumor necrosis factor-alpha (TNF-a) levels following exposure to an inflammatory stimulus (37). The exercise-induced elevation in IL-6 levels and simultaneous reduction in TNF-a levels suggest the potential therapeutic role of exercise in managing low-grade inflammation, which can exacerbate CKD. To the best of our knowledge, this is the first study to report the complex interplay between prolonged working hours, PA, and eGFR.

There were several inherent limitations in the design of this study. Firstly, its cross-sectional nature precludes the establishment of a causal relationship between exposure and health outcomes. It is plausible that individuals with pre-existing kidney issues may reduce their work hours to alleviate symptoms, potentially leading to an underestimation of the actual risk. Additionally, participants with lower kidney function may reduce working hours, leading to reverse causality. Thus, to ascertain causality, further investigation is warranted through longitudinal studies. Secondly, despite efforts to control for confounding variables, we cannot exclude the influence of other factors such as family history of kidney disease, meat consumption of analgesic medications, job-related stress, and occupation, which could impact the relationship between work hours and kidney function. For example, occupations with manual labor, especially those in high temperature environment, were more likely to develop CKD (38, 39). Higher processed meat consumption was associated with lower kidney function (40). Thirdly, our reliance on a single eGFR measurement introduces the possibility of misclassification of kidney function. According to the CKD definition, abnormalities in kidney structure or function should persist for at least 3 months. However, since our data were collected at a single time point, it was unable to confirm CKD cases according to this definition, potentially leading to the inclusion of patients with acute kidney injury. Forth, evaluating PA through questionnaire was not accurate enough. Additionally, the occupational PA was not collected in the NHANES data bank, resulted that the assessment of physical activity intensity exposure was incomplete. Additionally, varying impacts of different types and intensities of PA was not considered in the analyses. It would be more accurate to collect information through wearing an electronic bracelet.

Despite these limitations, the study possesses several strengths. Firstly, it is the first of its kind to investigate the relationship of kidney function with work hours and physical activity. Secondly, this study considered three criteria for prolonged working hours, yet the results remained robust. This underscores the urgency of addressing health concerns related to long working hours.

## 5 Conclusion

Overall, our findings underscore the importance of considering both PA levels and working hours in strategies aimed at preserving kidney function and preventing CKD. Encouraging individuals to engage in regular PA can promote overall health but not help offset the negative association of prolonged working hours on kidney function. This study can have implications for clinical and policy – oriented perspectives. This study may have implications for both clinical practice and policy development of labor. Future longitudinal studies are warranted to further elucidate the causal relationships of kidney function PA and working hours, as well as to identify potential mechanisms underlying these associations.

## Data Availability

The original contributions presented in the study are included in the article/**Supplementary Material**. Further inquiries can be directed to the corresponding authors.
