# Supplementary material for: The association of physical activity with kidney function risk among adults with long working hours
Source: Front Endocrinol (Lausanne). 2024 Sep 30;15:1415713. doi: 10.3389/fendo.2024.1415713 (PMC11471621; doi:10.3389/fendo.2024.1415713)
Supplement: Supplementary file 1 [file Table1.docx]

**Table S1.** Estimated association of CKD across long working hours performed by multiple logistic regression (*OR*, 95% *CI*).

|  | **Crude model** | **Model 1** | **Model 2** |
| --- | --- | --- | --- |
| **Weekly working hours** |  |  |  |
| ≤ 40 h/wk | Ref. | Ref. | Ref. |
| > 40 h/wk | 0.998 (0.783, 1.271) | 1.056 (0.823, 1.355) | 1.201 (0.937, 1.310) |
| **Weekly working hours** |  |  |  |
| ≤ 48 h/wk | Ref. | Ref. | Ref. |
| > 48 h/wk | 0.964 (0.735, 1.264) | 1.182 (0.926, 1.509) | 1.020 (0.771, 1.348) |
| **Weekly working hours** |  |  |  |
| ≤ 30 h/wk | Ref. | Ref. | Ref. |
| 31-40 h/wk | 1.038 (0.753, 1.432) | 1.065 (0.766, 1.481) | 1.047 (0.752, 1.457) |
| 41-48 h/wk | 1.076 (0.705, 1.644) | 1.112 (0.722, 1.715) | 1.048 (0.679, 1.619) |
| 49-54 h/wk | 1.072 (0.674, 1.705) | 1.174 (0.730, 1.888) | 1.118 (0.694, 1.802) |
| ≥ 55 h/wk | 0.952 (0.639, 1.420) | 1.056 (0.698, 1.599) | 1.018 (0.671, 1.543) |

Note. Ref. Reference. Model 1. Sex, age, race/ethnicity, and BMI were adjusted. Model 2. Smoking & alcohol habits, and hypertension were further adjusted.

**Table S2.** Estimated association of CKD across different weekly working hours category by physical activity time (*OR*, 95% *CI*).

| **Physical activity time** | **Participants *n* (%)** | ***OR* (95% *CI*)** | ***p*-value** |
| --- | --- | --- | --- |
| **< 150 min/wk** |  |  |  |
| ≤ 40 h/wk | 7352 (62.5) | Ref. | — |
| > 40 h/wk | 4407 (37.5) | 1.193 (0.884, 1.609) | 0.248 |
| **≥ 150 min/wk** |  |  |  |
| ≤ 40 h/wk | 4119 (61.7) | Ref. | — |
| > 40 h/wk | 2553 (38.3) | 0.690 (0.707, 1.015) | 0.123 |
| **< 150 min/wk** |  |  |  |
| ≤ 48 h/wk | 8897 (75.7) | Ref. | — |
| > 48 h/wk | 2862 (24.3) | 1.156 (0.825, 1.620) | 0.400 |
| **≥ 150 min/wk** |  |  |  |
| ≤ 48 h/wk | 5033 (75.4) | Ref. | — |
| > 48 h/wk | 1639 (24.6) | 0.775 (0.467, 1.285) | 0.322 |
| **< 150 min/wk** |  |  |  |
| ≤ 30 h/wk | 2141 (18.2) | Ref. | — |
| 31-40 h/wk | 5211 (44.3) | 1.175 (0.810, 1.705) | 0.395 |
| 41-48 h/wk | 1545 (13.1) | 1.342 (0.797, 2.259) | 0.269 |
| 49-54 h/wk | 1156 (9.8) | 1.319 (0.755, 2.302) | 0.331 |
| ≥ 55 h/wk | 1664 (14.5) | 1.350 (0.822, 2.218) | 0.236 |
| **≥ 150 min/wk** |  |  |  |
| ≤ 30 h/wk | 1353 (20.3) | Ref. | — |
| 31-40 h/wk | 2766 (41.5) | 0.739 (0.355, 1.539) | 0.419 |
| 41-48 h/wk | 914 (13.7) | 0.535 (0.230, 1.245) | 0.147 |
| 49-54 h/wk | 681 (10.2) | 0.703 (0.271, 1.823) | 0.469 |
| ≥ 55 h/wk | 958 (14.4) | 0.499 (0.220, 1.131) | 0.096 |

Note. ^*^ *p*-value < 0.05. Ref. Reference. Sex, age, race/ethnicity, BMI, Smoking & alcohol habits, and hypertension were adjusted.
